# Special Issue: Genetic Perspectives in Thyroid Cancer

**DOI:** 10.3390/genes12020126

**Published:** 2021-01-20

**Authors:** Susana Nunes Silva

**Affiliations:** Centre for Toxicogenomics and Human Health (ToxOmics), Genetics, Oncology and Human Toxicology, NOVA Medical School, Faculdade de Ciências Médicas, Universidade Nova de Lisboa, 1169-056 Lisboa, Portugal; snsilva@nms.unl.pt

Thyroid cancer is not just a common type of cancer, it is the most frequently diagnosed endocrine malignancy worldwide. The aetiology of thyroid cancer is essentially multifactorial and radiation is the best documented risk factor related to the disease. However, many questions are still open and seeking clarification, especially regarding the genetic aspects of this pathology, with clear implications not only on a scientific basis of the disease, but foremost in clinical oncology.

Thyroid gland tumours are a heterogeneous group of neoplasms that may arise from virtually any of the different cell types that are present in the thyroid gland. Although the malignant tumours are most frequently identified in thyroid follicular cells, it is clear that the molecular-genetic characterization is crucial and should be further explored to emphasize differences in tumour biological behaviours, aggressiveness, and disease prognostics. Papillary and follicular thyroid carcinomas (PTC and FTC, respectively) represent 85–90% and 5–10% of thyroid cancer cases, respectively, arising from thyroid follicular cells. These tumour histotypes retain their morphologic features and are often referred to as differentiated thyroid carcinoma (DTC). However, the aetiology of DTC is still unknown.

Considerable progress has been made in the understanding of thyroid carcinogenesis, in part based on retrospective and prospective studies published worldwide in recent decades, but also in the development of high-throughput approaches and the availability of improved diagnostic methodologies that lead to early diagnosis. In fact, over the last few decades, the research in thyroid cancer has highlighted the mutational landscape leading to better understanding of the molecular pathogenesis of DTC.

The hallmarks of cancer have been identified with several potential targets involved in gene expression deregulation, leading to the promotion of genetic instability and cancer development. The identification of genetic variants related to thyroid cancer has been a long way, though it is crucial to identify possible potential biomarkers of susceptibility. Some genetic variants have been identified in specific subtypes, which are related to the increased risk to develop this malignancy, such as the *BRAF* V600E mutation in PTC patients with a more aggressive phenotype if *TERT* promoter mutation coexists [1]. However, mutations in *RET* and *RAS* genes have also been identified as biomarkers in thyroid cancer patients. 

Alongside with genetic variants, epigenetic events and alterations in the expression of microRNAs (miRNAs) [2] and long noncoding RNAs (lncRNA) may also, through modulation of gene expression [3], drive the aberrant activation of oncogenic signalling pathways and the downregulation of thyroid-specific genes, thus contributing to the development, progression, and dedifferentiation of thyroid cancer [4].

Connected to this, the genetic instability inflicted in this malignancy, involving genetic and epigenetic alterations, might also compromise the treatment response. The standard treatment for thyroid cancer patients consists of surgical resection accompanied by post-thyroidectomy radioiodine (RAI) adjuvant therapy. The radioiodine therapy relies on the ability of 131I to be preferentially taken up in normal or neoplastic thyroid follicular cells. Its accumulation induces high DNA damage, which leads to cytotoxicity. However, the ionizing radiation does not only affect tumour cells, but the lesions may also impair normal cells. The most common lesions induced by radiation are double-strand breaks (DSBs), which are processed by enzymes involved in DNA repair pathways. The presence of genetic variants in these enzymes might impair the repair efficiency but also could influence the cytotoxic potential of RAI therapy, hence its efficacy in DTC treatment [5]. The development of induced DNA damage approaches can help in the evaluation of the efficacy of therapeutics as it has been shown to be an important tool for establishing biomarkers of susceptibility.

Although the most frequent variants identified in thyroid cancer contribute to sporadic disease, about 5 to 15% of all diagnosed cases are familial (first degree relatives) in nonmedullary thyroid cancer (NMTC), increasing to 25% in medullary thyroid cancer (MTC) cases. This adds to the clear existence of genetic predisposition factors related to this pathology [6]. Advances in molecular genetics and several candidate gene studies developed worldwide have linked the occurrence of this malignancy to some hereditary syndromes, some of which are related to germline mutations in *RET* genes.

Although thyroid cancer is not considered a common malignancy, with a 98% relative survival rate, this does not undermine the need to fully understand the mechanisms of disease in all of its aspects using all available tools from epidemiology approaches—genetic, epigenetic, regulation, therapeutic response, and resistance [7].

This collection aims to show how much has been achieved and what remains to be done with regard to thyroid cancer. Above all, bringing together researchers and clinicians is the key to better understand this heterogeneous disease.
